# 

**Published:** 1893-06

**Authors:** 


### Southern Medical Record

A Monthly Journal of Medicine and Surgery.

VOL. XXIII. ATLANTA, GA., JUNE, 1893. NO 6.

EDITORS:

A. W. GRIGGS, M. D.
WILLIS F. WESTMORELAND, M. D.
J. McFADDEN GASTON, M. D.
LOUIS H. JONES, M. D.
DAN. H. HOWELL, M. D., BUS. MGR.

Entered at the Postoffice at Atlanta, Ga., as Second-Class Matter.

Mutual Printing Company, Publishers, 27 East Hunter St., Atlanta, Ga.

